# The Dynamic Properties of a Brain Network During Spatial Working Memory Tasks in College Students With ADHD Traits

**DOI:** 10.3389/fnhum.2020.580813

**Published:** 2020-09-07

**Authors:** Kyoung-Mi Jang, Myung-Sun Kim, Do-Won Kim

**Affiliations:** ^1^Department of Psychology, Sungshin Women’s University, Seoul, South Korea; ^2^Department of Biomedical Engineering, Chonnam National University, Yeosu, South Korea

**Keywords:** attention deficit disorders with hyperactivity, working memory, brain oscillation, functional connectivity, graph theory

## Abstract

This study investigated deficits of spatial working memory in college students with attention-deficit/hyperactivity disorder (ADHD) traits using event-related potentials (ERPs) and the spatial 2-back task. We also computed sensory-level activity using EEG data and investigated theta and alpha neural oscillations, phase-locking values (PLV), and brain networks. Based on the scores from the Adult ADHD Self-Report Scale (ASRS) and Conners’ Adult ADHD Rating Scales (CAARS), an ADHD-trait group (*n* = 40) and a normal control group (*n* = 41) were selected. Participants were required to respond to whether the presented stimulus was at the same location as that presented two trials earlier. The ADHD-trait group showed significantly slower response times than the control group in the spatial 2-back task. In terms of spectrum, the ADHD-trait group showed significantly reduced theta power than the control group. In contrast, the ADHD-trait group exhibited an increased alpha power compared to the control group with the 250–1000 ms interval after stimulus onset. In terms of the PLV, the ADHD-trait group showed significantly weaker theta phase synchrony and fewer connection numbers in frontal-occipital areas than the control group. In terms of the theta brain network, the ADHD-trait group showed a significantly lower clustering coefficient and longer characteristic path length than the control group for the theta band. The present results indicate that college students with ADHD traits have deficits in spatial working memory and that these abnormal activities in neural oscillation, functional connectivity, and the network may contribute to spatial working memory deficits.

## Introduction

Attention-deficit/hyperactivity disorder (ADHD) is one of the most common neurodevelopmental disorders and is observed in around 3–5% of school-aged children (American Psychiatric Association [APA], 2013). However, recent studies have shown that 50–80% of individuals diagnosed with ADHD during childhood continue to exhibit inattention and impulsivity, even as adults (Asherson et al., 2016). Additionally, though the prevalence rate of ADHD in adults is estimated to be between 2.5 and 4.4% (Kessler et al., 2006; Simon et al., 2009; American Psychiatric Association [APA], 2013), it is reported that there are difficulties in diagnosing ADHD in adults due to the heterogeneous aspects of various diseases such as depression, anxiety, and drug use, as well as the severity of symptoms (McGough and Barkley, 2004; Mostert et al., 2015).

To date, no diagnostic biological markers of ADHD have been reported, despite various attempts to investigate its biological mechanisms (American Psychiatric Association [APA], 2013). Recently, neurophysiological measures such as electroencephalography (EEG) have been used to determine whether specific neurological abnormalities can be utilized to distinguish ADHD (Woltering et al., 2012; Lenartowicz et al., 2014; Michelini et al., 2019). Some studies suggest that neural oscillation, brain functional connectivity, or features from the brain network could distinguish ADHD patients from normal individuals (Ahmadlou et al., 2012; Mamah et al., 2013; Cao et al., 2014). Castellanos and Proal (2012) suggested that several neural networks, rather than specific brain regions, may affect the symptoms and cognitive deficits of ADHD.

Patients with ADHD have deficits in several cognitive areas, including attention, memory, and executive function. Of these, the deficit in working memory has received much attention (Hervey et al., 2004; McCabe et al., 2010). This is because working memory affects higher cognitive functions such as executive functions, and is reported to be associated with inattention, a typical symptom of ADHD (Gathercole et al., 2008; Kofler et al., 2010). Studies on adult ADHD patients and non-clinical individuals with ADHD traits have consistently reported a deficit in spatial working memory, which processes spatial information (Dowson et al., 2004; Kim et al., 2014; Mattfeld et al., 2015; Bollmann et al., 2017; Jang and Kim, 2018); however, the neurological mechanisms of the spatial working memory deficit in ADHD are not yet fully understood.

Electroencephalography studies reported that working memory is associated with theta and alpha oscillations (Klimesch et al., 2008; Kawasaki et al., 2010; Liu et al., 2016; Scharinger et al., 2017). In the theta band, power increase or event-related synchronization (ERS) have been observed to be relatively consistent in fronto-central and parietal regions while subjects performed working memory tasks (Jacobs et al., 2006; Jaiswal et al., 2010; Bickel et al., 2012; Lenartowicz et al., 2014). Theta has also been reported to be sensitive to searching for target stimuli and increasing cognitive load while processing working memory (Klimesch et al., 2000; Mazaheri and Picton, 2005; Khader et al., 2010). In the alpha band, power suppression or event-related desynchronization (ERD) is mainly observed over the parieto-occipital region during the maintenance of information (Pesonen et al., 2007; Itthipuripat et al., 2013; Dong et al., 2015). Alpha is also understood to play an important role in the inhibition of information irrelevant to the task (Manza et al., 2014; Roux and Uhlhaas, 2014). Studies examining the neurological mechanisms of working memory in patients with ADHD using *n*-back tasks or paradigms to investigate working memory levels, such as Sternberg and delayed-response sample tasks, have reported inconsistent results with either a decrease or increase in theta and alpha oscillations (Missonnier et al., 2013; Lenartowicz et al., 2014, 2016; Liu et al., 2016). Inconsistent results in previous studies may be attributed to the multidimensional nature of working memory (consisting of encoding, and storage and retrieval processes) and the clinical heterogeneity of ADHD (Pincham, 2014).

Some studies investigating functional connectivity and brain networks of working memory using EEG/MEG (magnetoencephalography) in healthy subjects observed the connectivity between frontotemporal, central-parietal, and occipital areas during working memory tasks (Chou et al., 2015; Wianda and Ross, 2019), and small-world network characteristics with a high clustering coefficient and low characteristic path length. These results imply that the brain regions involved in processing information consecutively while performing working memory tasks function efficiently (Shahabi et al., 2014; Dai et al., 2017). Studies using EEG on ADHD patients to investigate functional connectivity and brain networks in working memory are limited. Some studies on adults with ADHD have been reported using EEG/MEG to investigate brain networks while resting or performing cognitive control tasks (Franzen et al., 2013; Sudre et al., 2017; Michelini et al., 2019; Kiiski et al., 2020). However, explorative research is considered to be necessary, as no studies have been reported on functional connectivity and brain network related to working memory.

The severity of symptoms, the existence of comorbid disorders, and medication in adult ADHD patients are known to affect performance on working memory tasks, neuronal oscillations, and brain functional connectivity related to working memory. Thus, it has been suggested that studies should be conducted with non-clinical individuals with ADHD traits instead of with ADHD patients (Cubillo and Rubia, 2010; Konrad and Eickhoff, 2010; Cocchi et al., 2012; Liu et al., 2016). Moreover, some previous studies have observed that individuals with high-extreme ADHD traits have deficits in cognitive function similar to clinically diagnosed patients (e.g., response inhibition and interference control, working memory) (Polner et al., 2015; Kim and Kim, 2016; Jang and Kim, 2018). It has been suggested that these deficits may be caused by underlying neurological mechanisms such as differences in brain function, hence the necessity for such studies (Panagiotidi et al., 2018).

Therefore, the present study investigated the spatial working memory of non-clinical college students with ADHD traits using ERPs and spatial 2-back task. Graph theory were applied to examine the functional connectivity networks in different frequency bands. Specifically, while performing the spatial 2-back task: (1) theta and alpha bands were divided to investigate changes in neural oscillations and functional connectivity; and (2) clustering coefficient and characteristic path lengths were examined to determine whether college students with ADHD traits exhibited different network functions from those of the normal control group.

## Materials and Methods

### Participants

Details of the participants’ screening procedures are described in the previous study by our research group (Kim and Kim, 2016). Students with scores >4 on part A of the ASRS (Adult ADHD Self-Report Scale, Adler et al., 2003), >24 total points on the ASRS, and T scores >65 on the ADHD index of the CAARS (Conners Adult ADHD Rating Scale, Conners et al., 1999) were included in the ADHD-trait group (*n* = 40, 7 males and 33 females). Students with scores <3 on part A of the ASRS, <16 total points on the ASRS, and T scores <37 on the ADHD index of the CAARS, were included in the normal control group (*n* = 41, 11 males and 30 females) (see Supplementary file 1 for details). The internal consistency of the CAARS had a Cronbach’s alpha in the range of 0.74–0.95 (Christiansen et al., 2012), while the internal consistency of the ASRS had a Cronbach’s alpha of 0.89 (Kim et al., 2013). The two groups did not differ in age (21.35 ± 1.87 years for the ADHD-trait group, 21.58 ± 2.13 years for the control group, [*t*(79) = 0.53, ns]), educational level (14.80 ± 1.02 years for the ADHD-trait group, 14.66 ± 1.22 years for the control group, [*t*(79) = −0.57, ns]), or IQ scores (111.23 ± 9.70 scores for the ADHD-trait group, 113.88 ± 9.08 scores for the control group, [*t*(79) = −0.57, ns]).

The Structured Clinical Interview for Diagnostic and Statistical Manual of Mental Disorders, fourth edition (DSM-IV), non-patient version (SCID-NP) (First et al., 1996) was administered to ensure that participants did not have a history of psychiatric or neurological disorders, or alcohol/drug abuse. Interrater reliability coefficients of the SCID-NP were 0.7; the Korean version was administered in this study (Hahn et al., 2000). Additionally, the Korean Wechsler Adult Intelligence Scale (K-WAIS) was administered to measure participants’ intelligence quotient (IQ) (Yum et al., 1992). The internal consistency of the K-WAIS has been reported to range from 0.78 to 0.94 (Kim et al., 1992). Also, those who were left-handed or ambidextrous were excluded to control for the effect of brain lateralization. The participants provided written informed consent after receiving a description of the study and were paid for their participation. This study was approved by the Sungshin Women’s University Institutional Review Board (SSWUIRB 201-060).

### Spatial 2-Back Task

A spatial 2-back task was used to evaluate spatial working memory. This task consisted of two conditions: under the congruent condition, the locations of the current stimulus and stimulus presented two trials earlier were the same; under the incongruent condition, the locations of the current stimulus was different from that of the stimulus presented two trials earlier. A total of 360 trials (108 congruent trials, 252 incongruent trials) were randomly administered in two blocks. Participants were required to respond as fast and accurately as possible to the congruent stimuli by pressing a button with the index finger but to ignore the incongruent stimuli.

**FIGURE 1:**
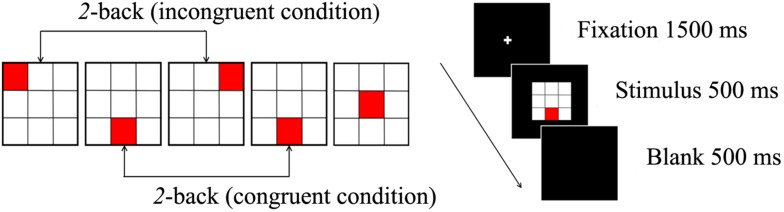
The spatial 2-back task consists of a congruent condition, incongruent condition (left), and the stimulus presented for 500 ms (right).

E-Prime software (version 1.2; Psychological Software Tools, Inc., Sharpsburg, PA, United States) was used to administer the spatial 2-back task. A crosshair was displayed for 1500 ms as a fixation point, then the stimulus was presented for 500 ms, and the inter-stimulus interval was 2500 ms. The distance between the participant and the computer monitor was 80 cm. We ensured that the distance for all participants was same by fixing the chair’s position. Before the experimental session, a block of 20 trials was administered to ensure that participants understood the instructions. The two types of conditions and the procedure for the stimulus presentation are illustrated in Figure 1.

### EEG Recording and Preprocessing

Electroencephalography was measured using 64-channel Geodesic Sensor Net connected to a 64-channel, high input impedance amplifier (Net Amp 300; Electrical Geodesics, Eugene, OR, United States) in a shielded and soundproofed experimental room. All electrodes were referenced to Cz, and impedance was maintained lower than 50 kΩ (Tucker, 1993). EEG activity was recorded continuously using a 0.1–100 Hz bandpass at a sampling rate of 250 Hz. Epoch contaminated by artifacts such as eye blinks and eye movement were removed based on the threshold of peak-to-peak amplitude ±100 uV from the electrodes (eye channels: channel 1, 5, 10, 17). The EEG data were segmented into 1500 ms epochs, including 500 ms pre-stimulus baseline period, and re-referenced to the average reference.

### Time–Frequency Analysis

In this study, event-related spectral perturbation (ERSP) was calculated to observe the power change over time during the spatial 2-back task. The spectral power of each band was calculated using a fast Fourier transform (FFT) along with a Hanning window. We averaged the results for two distinct frequency bands (theta: 4–7 Hz, alpha: 8–12 Hz) in four time-intervals (0–250 ms, 250–500 ms, 500–750 ms, and 750–1000 ms). We selected 20 non-adjacent electrodes (Fp1, Fp2, F7, F3, Fz, F4, F8, C3, Cz, C4, T3, T4, T5, T6, P3, Pz, P4, O1, Oz, and O2) out of 64 electrodes based on previous studies (Lachaux et al., 1999; Choi et al., 2016). ERSP was also calculated for areas of interest (AOIs) by averaging the power of designated channels: frontal (average of F3, Fz, F4), central (average of C3, Cz, C4), parietal (average of P3, Pz, P4), and occipital (average of O1, Oz, O2) areas. Only correct responses of the congruent conditions were included in the analysis. We used MATLAB version 8.3 (MathWorks, United States) for all analyses.

### Functional Connectivity Analysis

Single-trial signals were first transformed into narrowband signals in the theta (4–7 Hz) and alpha (8–12 Hz) bands through bandpass filtering. Then, the phase was extracted through Hilbert-transformation. The synchronization between each pair of selected channels was estimated in terms of phase-locking value (PLV) (Lachaux et al., 1999; Choi et al., 2016). To identify electrode pairs with significant phase synchrony, we used a double-threshold strategy based on two criteria (Kim et al., 2008; Choi et al., 2016). We determined that phase synchronization observed during performance of the spatial 2-back task increased significantly through the following criteria: (1) the significance level must be lesser than 0.05 when comparing the PLV during task execution with the PLV of surrogate data obtained from 200 random trial shuffles for each electrode pair; (2) the significance level must be lesser than 0.05 when comparing the PLV during task execution with the pre-stimulus baseline PLV. A statistical analysis of brain function connectivity was performed with only the values of phase synchronization that met both criteria.

### Graph Theoretical Analysis

The spatial pattern of inter-regional phase synchrony can be visualized based on graph theory. A network is composed of several nodes, connected to each other by edges. For statistical comparison of network characteristics, the clustering coefficient and characteristic path length were calculated using equations used in previous studies (Stam and Reijneveld, 2007). To determine whether the theta and alpha PLV patterns corresponded to a small-world network, which is the optimal structure for inter-regional communications, parameters in random networks were compared with the same number of nodes and edges as the network data obtained in the present experiment. That is, a random network of experimental data obtained in this study was computed, and then the clustering coefficient and characteristic path length of the random network were compared with the network characteristics of the experimental data (Watts and Strogatz, 1998). Small-world network index was present if >1, it means that it has the property of a small-world network (Humphries and Gurney, 2008).

### Statistical Analysis

#### Demographic Characteristics and Behavioral Results of the Spatial 2-Back Task

Demographic characteristics and scores on the ASRS and CAARS were analyzed using an independent *t*-test. Response times on the spatial 2-back task were analyzed with an independent *t*-test, and accuracy was evaluated with a mixed-design analysis of variance (ANOVA) with stimulus condition (congruent and incongruent) as the within-subject factors, and group (ADHD-trait and control) as the between-subjects factor. Only correct responses were included in the behavioral analysis.

#### Correlation Analysis Between EEG Measures and Behavioral Measures, and ADHD Symptomatic Scores

Possible correlations between EEG measures (including ERSP, PLV, Network) and behavioral measures, and ADHD symptoms, were explored within each group using the Pearson correlation coefficient.

## Results

### ADHD Questionnaire Measures

The demographic characteristics and scores on the ASRS and CAARS of the control and ADHD-trait groups are presented in Table 1. The ADHD-trait group had significantly higher part A scores, total scores of ASRS, and scores on the ADHD index of CAARS compared with the control group (*p* < 0.001).

**TABLE 1:** Scores on the ASRS and CAARS of control and ADHD-trait groups.

	**Control group (*n* = 41)**	**ADHD-trait group (*n* = 40)**	***t***
		
	**Mean (SD)**	**Mean (SD)**	
**ASRS (score)**			
Part A	0.61 (0.77)	4.70 (0.65)	−25.82***
Total points	12.05 (3.82)	44.43 (5.50)	−30.85***
**CAARS (score)**			
ADHD index	3.24 (1.80)	22.95 (2.24)	−43.69***
Inattention/memory	4.10 (3.27)	22.68 (5.44)	−18.57***
Hyperactivity/irritability	5.66 (2.70)	21.18 (6.34)	−14.28***
Impulsivity/emotional lability	2.90 (2.03)	21.18 (4.45)	−23.65***
Self-concept	3.00 (2.43)	12.15 (3.85)	−12.82***

*** indicates *p* < 0.001. ASRS, Adult ADHD Self-Report Scale; CAARS, Conners’ Adult ADHD Rating Scale; IQ, intelligence quotient; SD, standard deviation.

### Behavioral Results of the Spatial 2-Back Task

The mean response times and accuracy data for the two groups are presented in Table 2. Statistical analysis of response times under the congruent condition revealed that the ADHD-trait group showed significantly longer response times than the control group on the spatial 2-back task [*F*(1,79) = 13.15, *p* < 0.001, $\eta_{\text{p}}^{2}$ = 0.14]. In terms of accuracy, there was a significant main effect of condition [*F*(1,79) = 74.12, *p* < 0.001, $\eta_{\text{p}}^{2}$ = 0.48], such that the accuracy of the congruent condition was lower than that of the incongruent condition, whereas the two groups did not significantly differ under condition [*F*(1,79) = 0.22, *ns*, $\eta_{\text{p}}^{2}$ = 0.01].

**TABLE 2:** Mean response times and accuracies on the spatial 2-back task in the two groups.

	**Control group (*n* = 41)**		**ADHD-trait group (*n* = 40)**	
	**Congruent**	**Incongruent**	**Congruent**	**Incongruent**
Response time (ms)	431.87 (62.69)		492.49 (86.21)	
Accuracy (%)	88.07 (12.70)	99.41 (0.87)	87.10 (11.64)	99.10 (1.32)

*() indicates standard deviation.*

### ERSP in the Theta and Alpha Band

**FIGURE 2:**
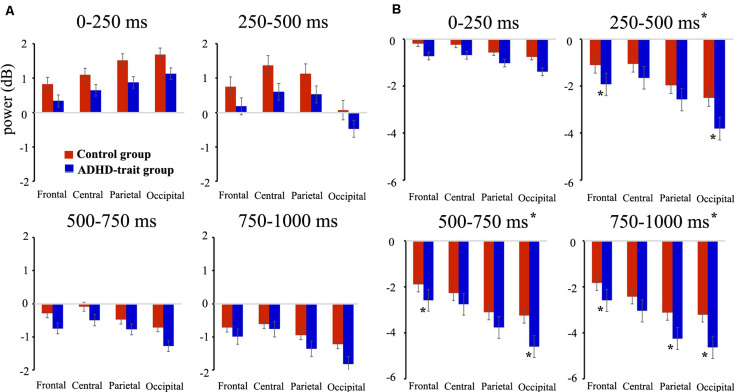
Comparison of mean absolute power for theta (4–7 Hz) **(A)**, alpha (8–12 Hz) **(B)** bands between the control group (red bar) and ADHD-trait group (blue bar). The ADHD-trait group exhibited a significantly lower theta power (results of the main effects of group) and higher alpha power (250–1000 ms intervals, results of a time interval × AOI × group interaction) than the control group (error bar: standard error, *p < 0.05).

With the theta band, we found the main effects of group, time interval and AOIs (see Table 3). The ADHD-trait group exhibited a significantly lower theta power than the control group. The greatest theta power was with the 0–250 ms interval, and the smallest was observed with the 750–1000 ms interval. With respect to AOI, theta power was highest in the central area, and the smallest was observed in the occipital area. An interaction effect of time interval × AOI was observed (see Table 3). A simple effect analysis revealed that the highest theta power was observed in the occipital area with the 0–250 ms interval [*F*(2.64,208.63) = 30.62, *p* = 0.001, $\eta_{\text{p}}^{2}$ = 0.28], whereas the highest theta power was observed in the central area with the 250–500 ms [*F*(2.60,205.51) = 52.27, *p* = 0.001, $\eta_{\text{p}}^{2}$ = 0.40], 500–750 ms [*F*(2.45,193.15) = 16.10, *p* = 0.001, $\eta_{\text{p}}^{2}$ = 0.17], and 750–1000 ms [*F*(2.52,198.81) = 29.90, *p* = 0.001, $\eta_{\text{p}}^{2}$ = 0.28] intervals. Figure 2A shows the mean theta powers in both control and ADHD-trait groups for all time intervals.

**TABLE 3:** ANOVA results with the factors group × time interval × AOI.

	**Theta**			**Alpha**		
	***F***	***p***	**$\eta_{\text{p}}^{2}$**	***F***	***p***	**$\eta_{\text{p}}^{2}$**
Group	6.34	0.01	0.07	5.27	0.05	0.06
Time interval (TI)	279.06	0.001	0.78	215.55	0.001	0.73
TI × group	1.01	0.38	0.01	1.54	0.22	0.02
AOI	18.89	0.001	0.19	52.65	0.001	0.40
AOI × group	0.28	0.81	0.01	2.28	0.11	0.03
TI × AOI	51.70	0.001	0.40	27.99	0.001	0.26
TI × AOI × group	1.65	0.13	0.02	2.36	0.05	0.03

*AOI, area of interest.*

Analysis of the alpha band revealed the main effects of group, time interval, and AOIs (see Table 3). The ADHD-trait group showed significantly higher alpha power than the control group. In addition, a time interval × AOI × group interaction was observed (see Table 3). The ADHD-trait group exhibited significantly higher alpha power than the control group in the frontal and occipital areas with the 250–500 ms [*F*(2.28,180.28) = 2.55, *p* = 0.05, $\eta_{\text{p}}^{2}$ = 0.03] and 500–750 ms [*F*(1.96,154.66) = 2.81, *p* = 0.05, $\eta_{\text{p}}^{2}$ = 0.03] intervals. With the 750–1000 ms interval, alpha power in the ADHD-trait group was significantly higher than in the control group in the frontal, parietal, and occipital areas [*F*(2.03,160.03) = 2.91, *p* = 0.05, $\eta_{\text{p}}^{2}$ = 0.04]. Figure 2B shows the mean alpha powers in both the control and ADHD-trait groups with all the time intervals.

### Correlations Between ERSP Activity and Behavioral Measure

The correlation between frequency band activity and behavioral measures was investigated within each group. Correlation analyses were applied only to frequency bands and behavioral response time that showed significant differences between the two groups. In the 250–500 ms interval in the control group, but not in the ADHD-trait group, a negative correlation between theta power and behavioral response time was found in the central [*r* = −0.31, *p* < 0.05] and parietal [*r* = −0.32, *p* < 0.05] areas. With the alpha band, both groups showed no significant correlation between the alpha power and the behavioral response time.

### Correlations Between ERSP Activity and ADHD Symptom

The correlation between frequency band activity and symptom severity scores was investigated in the ADHD-trait group. During the 0–250 ms interval, a negative correlation was found between the theta power and the self-concept score on the CAARS [*r* = −0.32, *p* < 0.05] at the parietal area, and between the theta power and the impulsivity/emotional lability score on the CAARS [*r* = −0.33, *p* < 0.05] at the occipital area. Additionally, a negative correlation between the theta power and the self-concept score on the CAARS [*r* = −0.38, *p* < 0.05] was found at 750–1000 ms in the occipital area. With the alpha band, a negative correlation between the alpha power and the self-concept score on the CAARS [*r* = −0.37, *p* < 0.05] was observed at 500–750 ms in the occipital area.

### Brain Functional Connectivity

**FIGURE 3:**
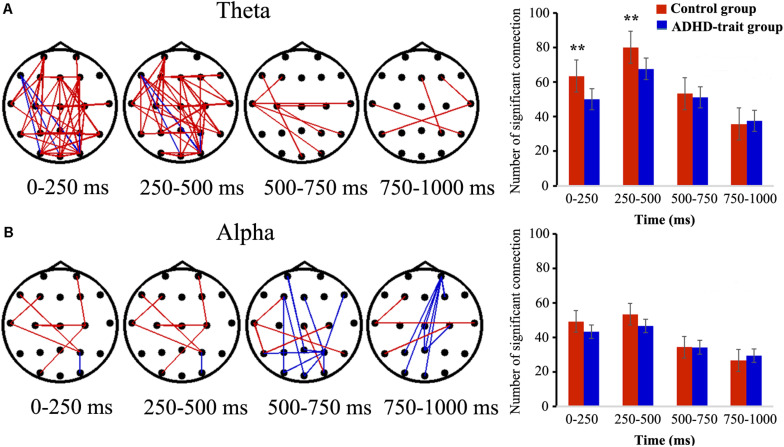
Spatiotemporal pattern of TPLVs **(A)** and APLVs **(B)**. Red: the control group has a significantly higher connectivity strength than the ADHD-trait group; blue: the ADHD-trait group has a significantly higher connectivity strength compared with the control group (left panels) (*p* < 0.05). The right panels show the time courses of the mean number of significant connections between the two groups (error bar: standard error, **p < 0.01).

Figure 3 shows the temporal variation of the inter-regional theta band PLVs (TPLVs, Figure 3A) and alpha band PLVs (APLVs, Figure 3B), and the mean number of significant connections between the two groups. The red line indicates that the control group had significantly higher connectivity strength than the ADHD-trait group, and the blue line indicates that the ADHD-trait group had significantly higher connectivity strength compared with the control group (*p* < 0.05).

The main effects of group [*F*(1,79) = 6.17, *p* < 0.05, $\eta_{\text{p}}^{2}$ = 0.07] and time interval [*F*(2.43,191.73) = 86.87, *p* < 0.001, $\eta_{\text{p}}^{2}$ = 0.52] were observed in terms of TPLV. With respect to group, the number of TPLV connections was significantly lower in the ADHD-trait group. The interaction effect of time interval × group was observed [*F*(2.43,191.73) = 5.38, *p* < 0.01, $\eta_{\text{p}}^{2}$ = 0.06]. In other words, with the 0–250 ms and 250–500 ms intervals, the number of TPLV connections was significantly lower in the ADHD-trait group than in the control group, whereas no significant difference was shown with the 500–1000 ms interval. In addition, the TPLV connections and behavioral measures within each group were not significantly correlated in the 0–250 ms and 250–500 ms intervals.

For the number of APLV connections, no significant main effect of group [*F*(1,79) = 1.31, *ns*, $\eta_{\text{p}}^{2}$ = 0.02], and no interaction effect of time interval × group were observed [*F*(2.26,178.54) = 1.88, *ns*, $\eta_{\text{p}}^{2}$ = 0.02].

### Graph Theoretical Analysis

**FIGURE 4:**
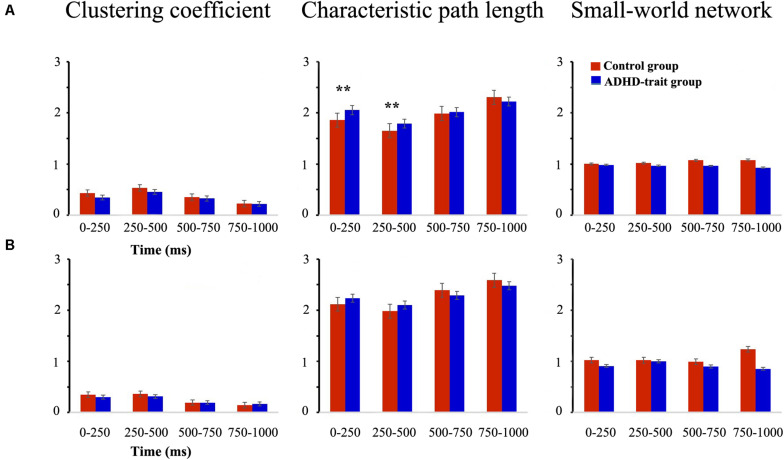
Network parameters of the theta **(A)** and alpha **(B)** bands between the control (red bar) and ADHD-trait group (blue bar). (a) Clustering coefficient, (b) Characteristic path length, (c) small-world network index (error bar: standard error, **p < 0.01).

The small-world network characteristics were evaluated using two representative graph theoretical measures: clustering coefficient and characteristic path length. Figure 4A shows the mean clustering coefficient, mean characteristic path length, and small-world network index in the control group and ADHD-trait group for the theta band. Analysis of the clustering coefficient revealed that the ADHD-trait group has a significantly lower clustering coefficient than the control group [*F*(1,79) = 8.08, *p* < 0.01, $\eta_{\text{p}}^{2}$ = 0.09]. In terms of characteristic path length, an interaction effect of time interval × group was observed [*F*(2.38,187.63) = 5.02, *p* < 0.01, $\eta_{\text{p}}^{2}$ = 0.06]. That is, with the 0–250 ms and 250–500 ms intervals, the ADHD-trait group exhibited a significantly longer characteristic path length than the control group. Also, the control group showed small-world network properties for all time intervals, whereas the ADHD-trait group did not show small-world network properties for all time intervals [*F*(1,79) = 5.02, *p* < 0.05, $\eta_{\text{p}}^{2}$ = 0.06]. If the small-world network index >1, it means it has a small-world network property. Pearson correlation analysis was performed to investigate the relationship between network parameters and ADHD symptoms in the ADHD-trait group. With the 250–500 ms interval, a positive correlation between the theta band clustering coefficient and ADHD index score on the CAARS was observed, [*r* = 0.34, *p* < 0.05], whereas no correlation was found between theta band characteristic path length and ADHD symptoms.

Figure 4B shows the mean clustering coefficient, mean characteristic path length, and small-world network index in the control group and ADHD-trait group for the alpha band. In terms of alpha band, the two groups did not significantly differ under the clustering coefficient [*F*(1,79) = 1.46, *ns*, $\eta_{\text{p}}^{2}$ = 0.02] and characteristic path length [*F*(1,79) = 0.02, *ns*, $\eta_{\text{p}}^{2}$ = 0.00]. In addition, the control group showed small-world network properties with the 0–250 ms, 250–500 ms, and 750–1000 ms intervals. However, the ADHD-trait group did not show small-world network properties with all the time intervals.

## Discussion

This study used ERPs and a spatial 2-back task to investigate whether abnormalities in neural activity and functional brain networks affect spatial working memory in college students with ADHD traits. Compared to the control group, the ADHD-trait group exhibited significantly longer response times to the congruent stimuli on the spatial 2-back task. In response accuracy, no significant difference was observed between ADHD-trait and control groups. These results are consistent with previous studies examining working memory in patients with ADHD (Missonnier et al., 2013; Kim et al., 2014; Lenartowicz et al., 2016; Gu et al., 2017) and suggest that adult ADHD patients need more time to perform a working memory task successfully than patients in the control group. In addition, the results might imply that the ADHD-trait group exhibits difficulties in spatial working memory.

### Neural Oscillation Characteristics

The ADHD-trait group showed significantly decreased theta power compared to the control group during the spatial working memory task. In addition, an increase in theta power was observed in the occipital area with the 0–250 ms interval and in the central area with the 250–1000 ms interval, which is consistent with previous studies on ADHD patients (Tsoneva et al., 2011; Missonnier et al., 2013). Previous findings suggest that theta band activity plays an important role in various cognitive tasks. Theta observed in the central area is related to decision-making (Jacobs et al., 2006), and theta observed in the occipital area is understood to be related to the visual attention required for visual working memory tasks (Awh and Jonides, 2001; Kawasaki and Yamaguchi, 2012). Accordingly, the present results suggest that the ADHD-trait group may have taken more time to process stimuli due to allotting insufficient attention to processing visuospatial information and having difficulty in task-related decision-making. This interpretation is supported by the correlation analyses in the present study, which show a negative correlation between faster response times and larger theta power. A significant negative correlation between theta power and response time was observed from the control group in the central and parietal regions with 250–500 ms post stimulus, while the correlation was not significant for the ADHD-trait group. That is, a faster response time was associated with increased theta power, which is consistent with previous studies that reported significant correlations between reaction time and theta power (Tsoneva et al., 2011). These results suggest that theta power is related to the performance of spatial working memory and can be used as a physiological indicator of spatial working memory.

In the case of an alpha band, the ADHD-trait group showed significantly increased alpha power compared to the control group with 250–1000 ms post stimulus. In particular, differences between the ADHD-trait and control groups were observed at the frontal and occipital areas, which is consistent with some previous studies on ADHD patients. For example, Missonnier et al. (2013) reported that adult ADHD patients exhibited significantly larger alpha power on the *n*-back task compared to a normal control group. Lenartowicz et al. (2014) also reported that children with ADHD showed significantly increased alpha power in occipital areas during the maintenance period in spatial working memory compared to a normal control group. The alpha power observed post stimulus presentation, i.e., during the maintenance period, is understood to be involved in maintaining the information of previously encoded stimuli (Klimesch, 1997; Klimesch et al., 2007) and suppressing task-irrelevant information (Manza et al., 2014; Chen and Huang, 2016; Liu et al., 2016). These results imply that patients with ADHD require more cognitive effort and time to maintain task-relevant information compared to a normal control group. Taken together with these previous studies, the increased alpha power in the ADHD-trait group observed in the present study suggests that individuals with ADHD traits require more cognitive effort to maintain task-related information and have difficulty with suppressing information not relevant to task performance, compared to the control group.

### Brain Functional Connectivity

We investigated functional connectivity and phase synchronization of theta and alpha bands to identify interactions between various brain regions during the performance of spatial working memory tasks. In the theta band PLV (TPLV), the ADHD-trait group showed significantly decreased functional connectivity across the anterior-posterior areas with 0–500 ms post-stimulus compared to the control group, as well as significantly fewer TPLV connections. Theta phase synchronization has been observed to be more spread across the brain than other frequencies (Fell and Axmacher, 2011). Accordingly, theta synchronization observed in various brain regions is suggested to have a function of integrating neural oscillation activity occurring in brain regions involved in working memory (Engel and Singer, 2001; Fell and Axmacher, 2011; Dai et al., 2017). Also, it has been reported that an increase in theta-related functional connectivity correlates with improvements in performance (Payne and Kounios, 2009; Burke et al., 2013; Toth et al., 2014; Akiyama et al., 2017). A study of theta functional connectivity during working memory tasks using EEG on ADHD patients has not yet been reported. However, some previous studies using fMRI to investigate functional connectivity between brain regions have reported that patients with ADHD have significantly lower functional connectivity in the prefrontal cortex, superior parietal area, cingulate cortex, and cerebellum. Moreover, significantly greater functional connectivity between the prefrontal cortex and intraparietal sulcus (IPS) has been reported in patients with ADHD compared to the healthy controls during the performance of working memory tasks (Wolf et al., 2009; Bedard et al., 2014; Wu et al., 2017). Considering the results of previous studies, the present results suggest that individuals with ADHD traits have brain dysfunctions based on the theta band observed in the frontal-occipital regions. That is, the results suggest that the ADHD-trait group lacks the ability to integrate information in brain regions related to spatial work memory, and it can be speculated that the abnormality in theta band connectivity can affect the deficits in spatial working memory. In the alpha band PLV (APLV), no difference in functional connectivity between the ADHD-trait group and control group was observed.

### Global Level and Local Level Network

This study investigated the functional separation and integration of the brain for efficient information processing using graph theory. One of the parameters of the network is the clustering coefficient that quantifies the functional segregation of the network, which is a measure of the local structure in the brain, indicating the proportion of neighboring nodes that are interconnected (Bosma et al., 2009). This means that the greater the clustering coefficient, the higher the local efficiency of information transmission in the network (Micheloyannis et al., 2006; Bullmore and Bassett, 2011; de Waal et al., 2014). The characteristic path length quantifies the functional integration of the network; the shorter the characteristic path length, the faster the information transmission speed of the network and the smoother the information processing (Watts and Strogatz, 1998; Micheloyannis et al., 2006; Rubinov and Sporns, 2010). We investigated the small-world characteristics of the brain network using a clustering coefficient and characteristic path length.

With the theta band, the ADHD-trait group showed a reduced clustering coefficient compared to the control group and increased characteristic path length with the 0–500 ms interval. Although studies on the functional brain network during a working memory task in patients with ADHD have not yet been reported, these results are consistent with the results of previous studies that reported abnormal functional brain networks during resting state and interference control tasks. For example, some studies investigating the characteristics of functional brain networks at resting state reported that patients with ADHD have higher local connectivity and lower global connectivity compared to the control group (Wang et al., 2009; Lin et al., 2014). It has also been observed that during an interference control task, patients with ADHD showed an increased clustering coefficient and characteristic path length compared to a normal control group (Liu et al., 2015). These findings suggest that the ADHD patient group is efficient in communicating information at the local area network level, but the ability to integrate information is inefficient compared to a control group (Ghaderi et al., 2017). Therefore, the present results suggest that college students with ADHD traits have the same abnormalities in functional brain networks observed in patients with ADHD. In addition, when performing the spatial 2-back task, these ADHD-trait individuals have difficulties both in transferring information at the local network level and integrating information at the global network level. With regard to the alpha band, no significant differences were observed between the groups in either clustering coefficient or characteristic path length; this suggests that college students with ADHD traits have a function similar to the control group in the alpha band.

### Relationship Between EEG and ADHD Symptoms Factors

We investigated the relationship between EEG measures and ADHD symptoms in the ADHD-trait group. A significant negative correlation was observed between theta power, alpha power and CAARS self-concept and impulsivity/emotional lability scale scores. Some previous studies have suggested that the ratio of two frequencies, such as the theta/beta ratio, controls some symptoms, such as impulsivity (Bluschke et al., 2016). Other studies have reported that neural oscillations observed during the encoding stage of the working memory task significantly predict ADHD symptoms (Lenartowicz et al., 2014). These results suggest that the decrease in theta power and increase in alpha power observed during the spatial work memory tasks are associated with ADHD symptoms (Missonnier et al., 2013). This study also identified the relationship between the characteristics of functional brain networks and ADHD symptoms in the ADHD-trait group. A significant positive correlation was observed between the clustering coefficient of theta and scores on the ADHD index of the CAARS in college students with ADHD traits. These results are consistent with the results of some previous studies that examined the relationship between structural or functional brain networks and ADHD symptoms in patients with ADHD (Missonnier et al., 2013). For example, Liu et al. (2015) reported a positive correlation between the clustering coefficient of beta observed during the performance of the interference control task, and the DSM-IV score indicating ADHD symptoms. The neural oscillations and functional brain network changes observed during the working memory task may suggest a relationship to ADHD symptoms; however, further research is needed.

### Study Limitations

Our study has several limitations that should be addressed in future research. First, only young adults participated in this study, which limits the generalizability of the present findings. Second, while the *n*-back task is useful for investigating working memory, it is limited to distinguish between information encoding and retrieval (Pinal et al., 2015). Therefore, further studies using the tasks capable of distinguishing different processing phases of working memory are needed. Last, this study analyzed the group difference in cognitive processing during a spatial working memory task. Future studies on resting EEG and its comparison with our results would provide more information on the spatial working memory deficits in ADHD.

## Conclusion

Our results suggest that individuals with ADHD traits exhibit deficits in spatial working memory, possibly due to abnormalities in neural oscillation, functional connectivity, and brain network. The study also showed that abnormalities in both theta and alpha oscillations are associated with working memory. For the theta, the ADHD-trait group exhibited significantly reduced power, weaker functional connectivity, lower clustering coefficient, and longer characteristic path length than the control group. These findings suggest that neural activity and networks based on theta oscillations play an important role in the deficits in spatial working memory in individuals with ADHD traits. Some studies suggest that neural oscillation, brain functional connectivity, or features from the brain network could distinguish ADHD patients from normal individuals (Ahmadlou et al., 2012; Mamah et al., 2013; Cao et al., 2014). Therefore, the results of this study indicate that theta oscillations could serve as a valuable index for diagnosis as well as deficits of spatial working memory in ADHD. In addition, present results could be used in establishing a treatment strategy such as neurofeedback, i.e., training an individual to control his/her neural oscillations, which further results in changes in the functional network of the brain over the long term (Marzbani et al., 2016).

## Data Availability Statement

The raw data supporting the conclusions of this article will be made available by the authors, without undue reservation.

## Ethics Statement

The studies involving human participants were reviewed and approved by the Sungshin Women’s University Institutional Review Board (SSWUIRB 201-060). The patients/participants provided their written informed consent to participate in this study.

## Author Contributions

K-MJ contributed to experimental design, data processing, analysis of results, and writing of manuscript. D-WK supervised the data analysis part of the experiment. K-MJ, M-SK, and D-WK revised the manuscript and carried out literature research. M-SK supervised aspects of the systematic literature review, preparation of the manuscript, revision, editing, and final intellectual content. All authors contributed to the article and approved the submitted version.

## Conflict of Interest

The authors declare that the research was conducted in the absence of any commercial or financial relationships that could be construed as a potential conflict of interest.
